# Improvement of a Three-Layered *in vitro* Skin Model for Topical Application of Irritating Substances

**DOI:** 10.3389/fbioe.2020.00388

**Published:** 2020-05-08

**Authors:** Freia F. Schmidt, Sophia Nowakowski, Petra J. Kluger

**Affiliations:** Reutlingen Research Institute, Reutlingen University, Reutlingen, Germany

**Keywords:** three-layered skin model, *in vitro* skin irritation testing, alternatives to animal testing, tissue engineering, subcutis

## Abstract

In the field of skin tissue engineering, the development of physiologically relevant *in vitro* skin models comprising all skin layers, namely epidermis, dermis, and subcutis, is a great challenge. Increasing regulatory requirements and the ban on animal experiments for substance testing demand the development of reliable and *in vivo*-like test systems, which enable high-throughput screening of substances. However, the reproducibility and applicability of *in vitro* testing has so far been insufficient due to fibroblast-mediated contraction. To overcome this pitfall, an advanced 3-layered skin model was developed. While the epidermis of standard skin models showed an 80% contraction, the initial epidermal area of our advanced skin models was maintained. The improved barrier function of the advanced models was quantified by an indirect barrier function test and a permeability assay. Histochemical and immunofluorescence staining of the advanced model showed well-defined epidermal layers, a dermal part with distributed human dermal fibroblasts and a subcutis with round-shaped adipocytes. The successful response of these advanced 3-layered models for skin irritation testing demonstrated the suitability as an *in vitro* model for these clinical tests: only the advanced model classified irritative and non-irritative substances correctly. These results indicate that the advanced set up of the 3-layered *in vitro* skin model maintains skin barrier function and therefore makes them more suitable for irritation testing.

## Introduction

The assessment of skin toxicity is an essential part of the analyzing the overall effect of chemicals and pharmaceutical products. As an ethical alternative for skin toxicity testing, and due to the limited transferability of results from animal assays to human reactions, *in vitro*-generated organ-like reconstructed human skin models have been developed using tissue engineering techniques. Global legislation has been committed to the development of alternative test methods, in accordance to the 3Rs (reduction, refinement, and replacement of animal experimentation) established by Russell et al. (1959).

Several skin models are currently commercially available from various producers and academic institutes. While, such models are used for skin corrosion, skin irritation, skin barrier formation, and skin absorption assays, amongst others, the only approved *in vitro* tests use epidermal models (OECD, 2019a, b). The absence of dermal and subcutaneous layers limits the application of the models, as well as the crosstalk of the three skin layers, the regulation of skin morphology, homeostasis, and metabolic activity (Maas-Szabowski et al., 1999; Oesch et al., 2014; Wiegand et al., 2014). Different artificial skin models constituting an epidermal and dermal layer are available, however, the subcutaneous part of the skin in most current models is neglected.

As an endocrine and paracrine organ, adipose tissue plays an important role in the irritating and sensitizing reaction of tissue and in the storage and metabolization of drugs. Therefore, this part of the skin is crucial for the assessment of effects of drugs on the skin and for the storage of various substances. Many studies confirm that especially lipophilic adipose tissue can absorb harmful substances highlighting the need for test systems to categorize such substances. The process of accumulation of a substance in tissue has so far been neglected in most experiments (*in vivo*, as well as *in vitro*). A three-layered skin model reflects the native skin more accurately, as well as extending the test spectrum with new test substances and further endpoints. First efforts to generate three-layered skin models have been made using stem cells, which were differentiated into the adipogenic lineage (Bellas et al., 2012). However, stem cells are time-, material-, and cost-intensive. Additionally, subcutaneous layers created using stem cells have so far lacked a sufficient amount of functional adipocytes. As an alternative, using mature adipocytes to create a functional subcutis (Huber et al., 2016b) offers promising properties. They can be isolated in large numbers and are fully functional without the need for further differentiation from adipose tissue. To build up artificial connective tissue, collagen hydrogels are commonly used as the main protein component of the extracellular matrix of the native dermis are collagen type I and type III (Rossi et al., 2015). However, fibroblasts contract collagen-based hydrogels in culture resulting in the epidermal layer losing the close connection to the insert wall, and eventually leading to the loss of skin barrier function (Ackermann et al., 2010). To overcome the fibroblast-mediated contraction of hydrogels, different approaches were used so far. A reduced contraction of collagen hydrogels was caused by physical and chemical modifications, for example using plastic compression or cross-linking of the collagen matrix (Braziulis et al., 2012; Lotz et al., 2017). However, in these cases, the contraction cannot be completely prevented, and the modifications further influence cell behavior. To overcome these shortcomings, in this study we explored altered culture conditions to maintain the epidermal barrier function in collagen-based skin models.

We aimed to improve the barrier function of our already established model using a three-layered *in vitro* skin model, including an epidermis, dermis, and a subcutis by adapting the culture conditions. The novel skin model overcomes an epidermal shrinkage by applying keratinocytes to the insert membrane which separates the epidermis from the underlying dermis. Using this functional epidermis, various barrier function tests were performed highlighting that the modified system is superior in terms of epidermal barrier function. Additionally, modified skin models with an advanced construction were morphologically characterized and compared to native human skin. Finally, we evaluated the applicability of the three-layered skin models for irritation studies by analyzing the irritation potential of known substances. This is essential for their application as an alternative to animal testing.

## Materials and Methods

### Human Tissue Samples

All research was carried out in accordance with the Declaration of Helsinki on human medical research. Patients gave written consent after being given information about the use of their probes. This as in accordance with the permission of the Landesärztekammer Baden-Württemberg (F-2012-078; for normal skin from elective surgeries).

### Cell Isolation and Culture

Adult mature adipocytes were isolated as described previously byHuber et al. (2016a). Human fatty tissue was provided by Dr. Ziegler (Klinik Charlottenhaus, Stuttgart) (Huber et al., 2016a).

Primary keratinocytes and fibroblasts were isolated from biopsies of human foreskin of surgeries performed by Dr. Z. Yurrtas from Stuttgart. Their isolation was performed according to protocols previously described by Huber et al. Keratinocytes were used in passage 3 for all experiments.

### Construction of Three-Layered Skin Models and Advanced Three-Layered Skin Models

The construction of three-layered skin models was based on the procedure previously described by Huber et al. (2016b) with some modifications. Three-layered skin models were composed in a collagen type I hydrogel (10 mg/mL from rat tail, Corning, United States) in 12-well plate inserts (Greiner Bio-One, 0.4 μm pore diameter). The subcutaneous layer, is made of collagen gel mixed with freshly isolated adipocytes and a gel neutralization buffer [10× DMEM/Ham’s F12 (Biochrom) and 50 mM NaOH in demineralized water (1:1) with 0.2 M NaHCO_3_ and 0.225 M HEPES (Serva Electrophoresis)] in a ratio of 4:4:1. 300 μL was pipetted into each insert and the gel run for 20 min at 37°C. The dermal layer consists of fibroblasts embedded in a collagen hydrogel. Collagen, fibroblast suspension and gel neutralization buffer were mixed in the same manner as described for the subcutaneous layer (ratio 4:4:1). Per skin model, 1.5 × 10^4^ fibroblasts in 300 μL collagen solution were seeded above the adipose tissue layer. Then the models were incubated for another 20 min at 37°C. Inserts were placed into deep-well plates containing 5 mL of adipocyte maintenance 1 medium (ZenBio) supplemented with 1.44 mM CaCl_2_ (Applichem) and 73 μg/mL ascorbic acid-2-phosphate (Sigma-Aldrich; =AM1-Air) and were incubated for 24 h. The next day, keratinocytes were obtained by accutase incubation (Sigma-Aldrich) for 20 min at 37°C and 5% CO_2_. On each insert, 5 × 10_5_ cells were seeded in 500 μL keratinocyte growth medium and allowed to attach for 1 h. Then 5 mL AM1-Air was added to the wells. After 24 h, the remaining medium in the insert was removed to allow airlift conditions for the keratinocytes.

For the advanced three-layered skin models, pieces of silicone tubing (1 cm width, 15 mm inner diameter, 2 mm wall thickness, cut into pieces of 20 mm height; Esska) were disinfected with 70% ethanol (Brenntag) and attached to the bottom of the cell culture inserts (Greiner Bio-One). Inserts were turned over and the dermal layer was pipetted into the piece of tubing, following the subcutaneous layer. Gels were prepared as described above for the previous three-layered skin model. After seeding, the inserts were turned over again and placed into a 12-deep-well plate containing 3 mL AM1-Air. After 24 h, keratinocytes were seeded onto the insert membrane and set to airlift culture after another 24 h. Three-layered skin models and advanced three-layered skin models were cultured for 15 days in AM1-Air until further evaluation.

### Measurement of Epidermal Area

The area of the epidermis was measured at days 4, 7, and 14 using Image J by comparison to a set scale.

### Determination of Skin Barrier by Topical Detergent Application

The barrier of skin and advanced skin models was tested by application of 1% Triton X-100 for 1.5 h at 37°C and 5% CO_2_. Remaining Triton X-100 was aspirated and the skin models were washed twice with Phosphate buffered saline (PBS). The viability of the skin models was measured via a WST-1 assay (Takara Clontech), with absorbance measurement of 200 μL sample at 450 and 620 nm. Values were normalized to non-treated skin models.

### Permeability Experiments With Fluorescent Molecules

The silicone tubes were removed from the inserts of the advanced skin models. Skin models and advanced skin models were placed into 12-well plates. AM1-Air medium (1 mL) was applied to the receiver compartment and 300 μL of fluorescent solution [1 mg/mL fluorescein isothiocyanate dextran (4 kDa, Sigma-Aldrich) in AM1-Air, 0.25 mg/mL fluorescein sodium (Roth) in AM1-Air] to the donor compartment. The plate was incubated at 37°C under agitation over 6 h. At defined time points (0.5, 1, 2, 4, 6 h), an aliquot (100 μL) was withdrawn from the receiver compartment and the removed liquid was replaced with AM1-Air medium. Fluorescence was measured by a micro-plate reader (Tecan) at 485/530 nm.

### Irritation Experiments

#### Study Design

The irritation tests were performed according to the OECD test guideline 439 (OECD, 2019b). The reference substances (Table 1) comprised one irritant (category 2 substance) according to the United Nations Globally Harmonized System of Classification and Labelling of Chemicals (UN GHS), and one non-irritant (no category substance). Two controls were included in each test run. Phosphate buffered saline (Lonza) was applied to the top of the models as a negative control (non-irritant) and a 5% aqueous solution of sodium dodecyl sulfate (SDS; Sigma-Aldrich) served as positive control (irritating). All substances and controls were tested on three skin model replicates per test run.

**TABLE 1:** List of two reference chemicals defined in the OECD performance standard Test No. 439 (*in vitro* Skin Irritation: Reconstructed Human Epidermis Test Method) to assess the predictive capacity of the skin models.

Test substances	Supplier	Physical state	UN GHS category
2-Propanol	Brenntag	Liquid, clear, colorless	No category (non-irritant)
Heptanal	Thermo Fisher Scientific	Liquid, clear, colorless	Category 2 (irritant)

#### Test Protocol

The skin irritation test was carried out according to the protocol published by Groeber et al. (2016) with some modifications. Briefly, 100 μL of the liquid test substances were applied to the skin models and advanced skin models. After a treatment time of 35 min at room temperature, the skin models were washed eight times with 600 μL PBS each and additionally immersed five times into 60 mL fresh PBS. After a post-exposure incubation of 42 h at 37°C and 5% CO_2_, tissue viability was assessed via a WST-1 assay (TaKaRa Bio Europe, France). For that, skin models were cut out of the inserts and incubated with 1 mL WST-1 reagent in AM1-Air (1:10) for 30 min at 37°C and 5% CO_2_. The WST-1 reduction was quantified by measuring the optical density at a wavelength of 450 and 620 nm using a micro-plate reader (Tecan). Tissue viability of treated models was normalized to the negative control (PBS), which was set to 100%.

#### Prediction/Evaluation Model

In this study, the prediction/evaluation model defined in the performance standards of the OECD test guideline 439 was used. A 50% threshold was used for the prediction/evaluation of skin irritation from viability measurements. A substance that reduced the average viability after the skin irritation test to below 50% was classified as “irritating” or “category 2” following the UN GHS system. Substances with mean viability above 50% were classified as “non-irritating” or “no category.”

#### Quantification of Extracellular Interleukin Concentration by ELISA

To investigate the cytokine response following skin irritation, the extracellular interleukin (IL) concentration of IL-1α, IL-6 and IL-8 was quantified by ELISA (PeproTech). This was carried out according to the manufacturer’s instructions. Briefly, supernatants were collected before and after the irritation test (42 h after the last medium change). Samples were diluted as follows: for IL-1α undiluted, for IL-8 1:50, and samples treated with 2-propanol 1:100, for IL-6 1:2 and samples treated with 1:10. For color development, 100 μL tetramethylbenzidine (TMB) substrate was added to each well. The reaction was stopped with 100 μL 1 M sulfuric acid (Thermo Fisher Scientific) and the absorption was measured at 450 nm with a wavelength correction of 620 nm (Tecan). The concentration of IL-1α, IL-6 and IL-8 was calculated using an evaluation excel sheet provided by PeproTech.

### Hemalaun-Eosin Staining and Immunofluorescence Staining

Skin models were fixed using 4% paraformaldehyde (Roth) for 4 h and watered with demineralized water for several hours. After embedding tissues in paraffin, sections of 5 μm were generated. Hemalaun-eosin staining was performed according to a standard staining procedure.

For immunofluorescence staining, tissue sections were deparaffined according to a standard protocol. Sections for filaggrin, cytokeratin 10 and 14 and vimentin were heat demasked with a target retrieval buffer pH 6 and Ki67 and perilipin A with a target retrieval buffer pH 9 for 20 min in a preheated steamer. Tissue sections were blocked with 3% BSA in 0.1% Triton X-100 for 30 min. Primary antibodies (cytokeratin 10: 1:200, Santa Cruz; cytokeratin 14: 1:1000; and filaggrin: 1:500, both Boster Biological Technologies; Ki67: 1:100; and vimentin: 1:1000, both Abcam; perilipin A: 1:500, Sigma-Aldrich) were diluted with blocking solution and incubated overnight at 4°C. Secondary antibodies Alexa Fluor 488 (1:500, Abcam) and Cy3 (1:250, Jackson ImmunoResearch Laboratories) were used for 1 h at room temperature. Sections were covered with Fluoromount-G containing DAPI (Life Technologies) and a coverslip and analyzed with a fluorescence microscope (Zeiss).

### Statistics

All experiments were repeated as indicated in figure legend The samples of three donors were examined. Data was compared using one-way analysis of variance (ANOVA) with repeated measurements and a mean value comparison according to Tukey using Origin Pro. Statistical significance was stated as ^∗^*p* < 0.05.

## Results

### The Advanced Construction of Skin Models Prevents Dermal-Mediated Contraction of the Epidermis

**FIGURE 1:**
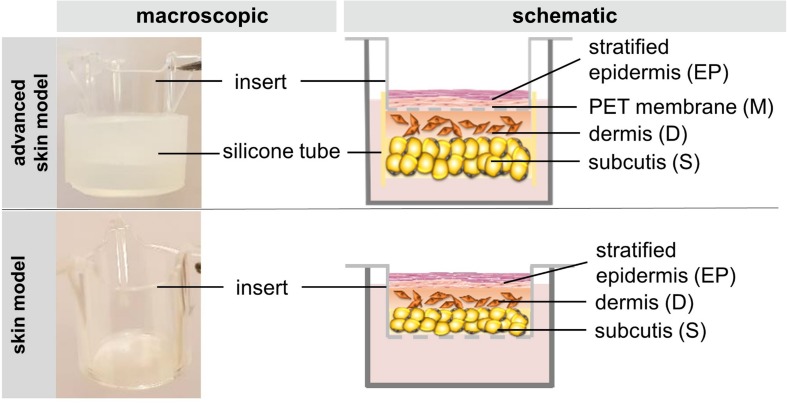
The figure schematically depicts the construction of the skin model and the advanced skin model.

To understand what prevents dermal-mediated contraction of the epidermal layer of skin models, the new construction approach was compared to skin models cultured with a more common method (Figure 1). Freshly isolated adipocytes and fibroblasts were encapsulated into a collagen hydrogel and seeded into a silicone tube attached beneath the cell culture insert. The next day, keratinocytes were seeded into the insert directly on the PET membrane, which functions as an artificial basal membrane. The modified setup differs from the previous structure in which all three skin layers are inserted directly into the cell culture insert without the separating membrane between epidermal and dermal part.

**FIGURE 2:**
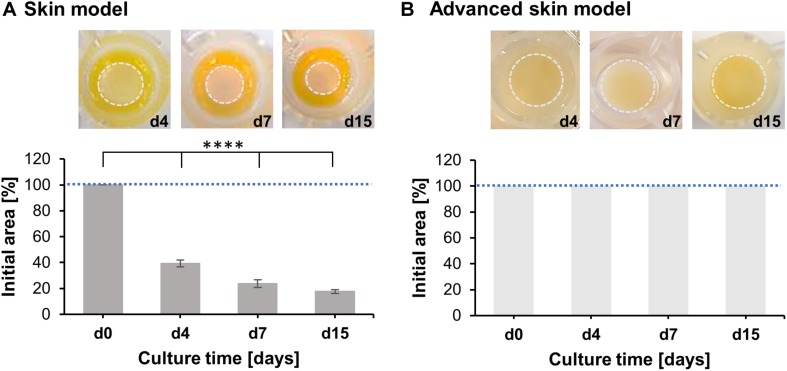
Analysis of skin model area over time. Pictures show representative tissue models after 4, 7, and 15 days of culture. Dashed circles (light gray) indicate the contracted area of the epidermal-dermal part. Bar graphs outline percentages of the initial area at the respective days normalized to the area at day 0. Mean values are plotted with SD. Dashed blue lines highlight 100% of the initial skin model area. **(A)** Evaluation of skin model area (*n* = 3), **(B)** Evaluation of advanced skin model area (*n* = 3), level of significance ^****^p < 0.0001.

To assess the capability of advanced skin models to resist cell-mediated contraction of the epidermal part, the epidermal layer was monitored periodically for 15 days (Figure 2). During the culture period, the epidermis of the common skin models continuously contracted by 17.68 ± 1.46% of the initial epidermal surface (Figure 2A). In opposition, no contraction was detected in the advanced skin models, whereby a surface area of 100% was retained (Figure 2B).

These observations suggest that the advanced skin models prevent dermal-mediated contraction of the epidermal layer, compared to more commonly used *in vitro* models.

### Advanced Skin Models Show Improved Skin Barrier Functions

**FIGURE 3:**
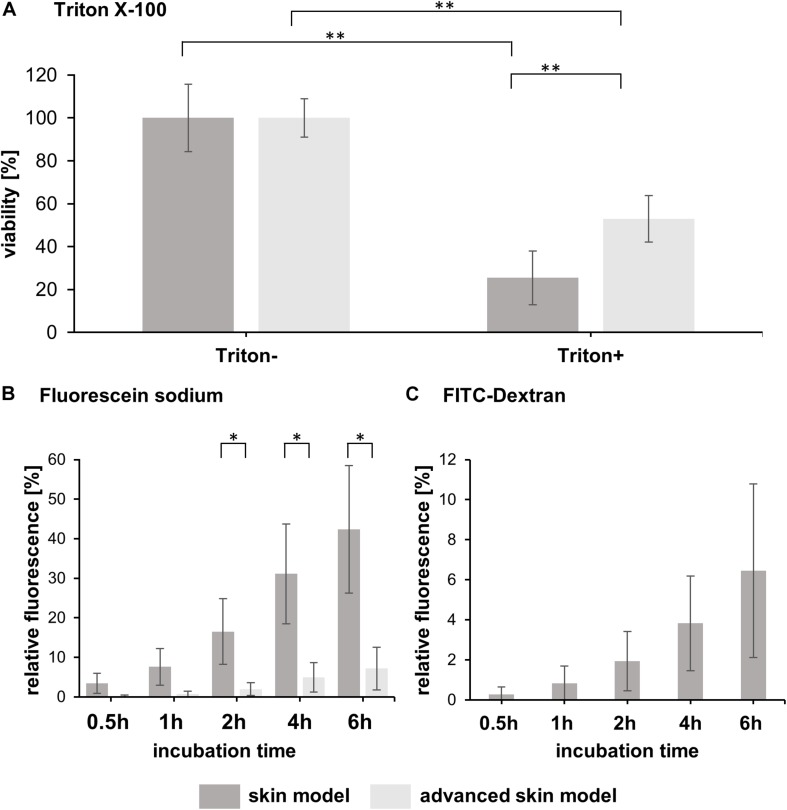
Analysis of skin model barrier function. **(A)** The bar graph shows the percentual viability of skin models and advanced skin models after treatment with (Triton+) and without a detergent (Triton-) (*n* = 3). The values were normalized to the untreated skin model/advanced skin model which was set to 100%. Level of significance ^∗∗^*p* < 0.01. **(B)** Fluorescein sodium and **(C)** FITC-dextran were applied topically to the skin models and permeability was assessed over a time period of 6 h with sampling at 0.5, 1, 2, 4, and 6 h after application. The bar graphs depict the percentage of relative fluorescence values normalized to a reference which was set to 100% (fluorescent stock solution in cell culture medium). For FITC-dextran, only values for skin models are shown, as the values for the advanced skin models were below the detection limit. Mean values are presented with SD (*n* = 3). Level of significance ^∗^*p* < 0.05.

The barrier integrity is the most integral to the skin models and ensures reliable testing. Therefore, the resistance to a detergence (Triton X-100) and permeability assays with fluorescent molecules (0.25 mg/mL fluorescein sodium and 1 mg/mL FITC-dextran) were conducted on day 15 of the advanced skin models (Figure 3).

The viability of the models after Triton application was significantly reduced by 25.43 ± 12.46% for common/traditional skin models and to 53 ± 10.82% for the advanced models, compared to the respective untreated models (Figure 3A). Moreover, the mean viability value of the advanced skin models treated with Triton was significantly higher than that of the more traditional skin models, indicating a higher cell survival in the advanced skin model following detergent application.

To assess permeability, fluorescein sodium and FITC-dextran were applied topically to the skin models and incubated for 6 h. Samples were taken after 0.5, 1, 2, 4, and 6 h of treatment. Fluorescence values were normalized to the reference/untreated samples and displayed as a percentage. With the fluorescein sodium treatment, no significant difference in the mean values between the skin model approaches was observed for the first two sampling times [0.5 h: 3.47 ± 2.55% (traditional skin model), 0.2 ± 0.24% (advanced skin model), 1 h: 7.59 ± 4.59% (traditional skin model), 0.72 ± 0.7% (advanced skin model)]. From 2 h of starting the treatment the fluorescence values of the skin model (16.53 ± 8.29%, 31.11 ± 12.63%, 42.39 ± 16.13%) were significantly higher compared to the advanced traditional skin model (1.94 ± 1.63%, 4.94 ± 3.75%, 17.17 ± 5.38%; Figure 3B).

For FITC-dextran, the advanced skin models showed no measurable changes in permeability compared to the untreated. For the skin models, the relative fluorescence values were 0.28 ± 0.37% after 0.5 h of incubation, 0.83 ± 0.87% after 1 h, 1.94 ± 1.49% after 2 h, 3.83 ± 2.37% after 4 h and 6.45 ± 4.33% after 6 h (Figure 3C).

### Advanced Skin Models Recapitulate the Morphology of Human Skin

**FIGURE 4:**
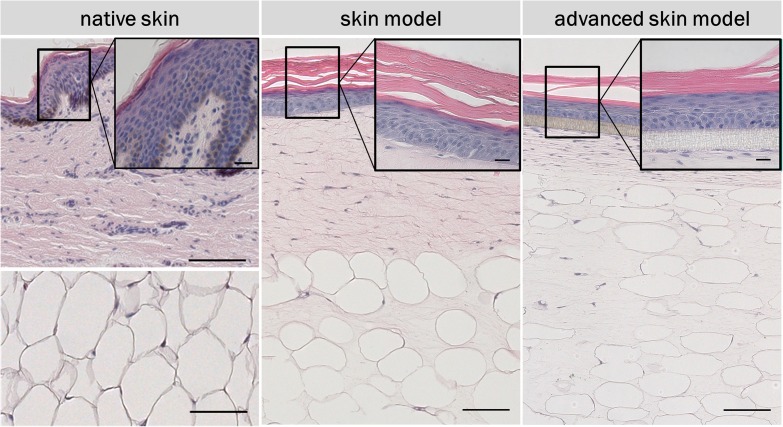
H&E staining of *in vivo* and *in vitro* skin. The epidermis contains all characteristic layers: basal, spinous, granular and cornified layer. The artificial basal lamina (insert PET membrane) is shown for the advanced skin model. Cell nuclei are stained in purple/blue and plasma proteins, collagen, and keratins are stained in pink. Black arrows point at nuclei. Scale bars: 100 μm (low magnification), 20 μm (high magnification).

To analyze the basic morphological features of the traditional skin models, H&E staining were conducted and compared to human skin samples. Both *in vitro* skin models formed a multilayered epidermis, a dermal compartment, and a subcutaneous layer (Figure 4). The architecture of the skin models and human skin were comparable. In the lower part of the epidermis, several layers of living cells can be identified both in the skin models and the human skin. These formed a defined stratum basale, granulosum, and spinosum. The epidermal keratinocytes showed a cuboid morphology typical of human keratinocytes in the basal layer. In both skin models, the stratum granulosum consisted of flat cells with certain cells lacking a nucleus. The stratum spinosum was also comparable the *in vitro* models and the human skin. However, human skin and skin models differed in the thickness of the stratum spinosum. This appeared to be regular in the skin models compared to the irregular thickness of the native model. This was due to the papillary bodies of the human skin. In the dermis of human skin, the fibroblasts were clustered whereas in the skin models fibroblasts were homogenously distributed throughout the dermal part and appeared more elongated and spindle-shaped as well as being less numerous. The underlying subcutis showed round-shaped adipocytes with detectable cell nuclei comparable to human adult adipose tissue. However, the subcutaneous layer of the skin models contained more intermediate matrix and the cells were not as densely packed as in the human adipose tissue.

**FIGURE 5:**
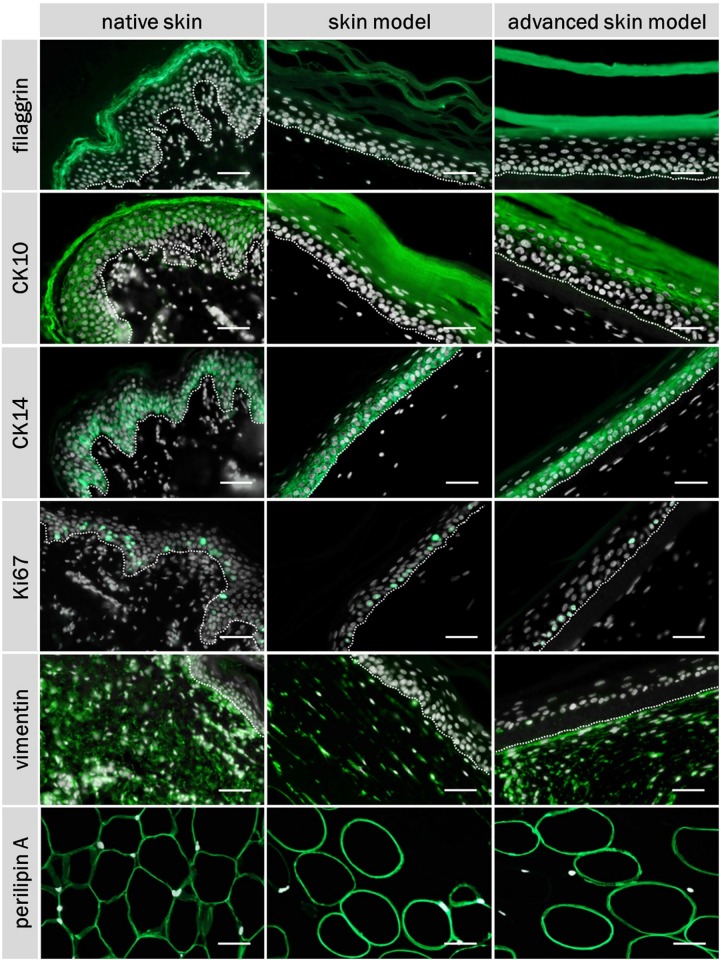
Immunofluorescence staining of filaggrin, CK10, CK14, Ki67, vimentin, perilipin A, and DAPI of native skin and skin models. Cell nuclei (stained with DAPI) are illustrated in white, characteristic proteins (stained with respective specific antibody) in green. White dashed line shows epidermal-dermal border. Scale bar: 50 μm.

The skin models were characterized using the three differentiation markers: Cytokeratin 10 and 14, and fillagrin (Figure 5). Ki67 staining was also carried out to assess cell proliferation in the epidermal layer (Figure 5). Human dermal fibroblasts were visualized via vimentin (Figure 5). Adipocytes of the subcutaneous layer were stained using the coating protein perlipin A (Figure 5). The co-localization of the epidermal differentiation markers cytokeration 10 and 14 and of filaggrin could be seen in the skin models as well as in human skin. Cytokeratin 10 was present as part of the intermediate filament network in the suprabasal layers, whereas cytkeratin 14 was most strongly represented in the basal layers. Fillagrin was found particularly in the stratum corneum. Proliferative cells were identified by Ki67 staining in the base layer of the epidermis, for both the skin models and human skin. The distribution of the skin fibroblasts in the skin portion of the models were identified by vimentin staining and comparable to the dermis of the human skin. The subcutis was visualized using the perlipin A coating protein. Both skin models showed adipocysts completely coated with perlipin A. This corresponds to the native fat tissue structure.

### Advanced Skin Models Are Suitable to Test the Irritation Potential of Substances

**FIGURE 6:**
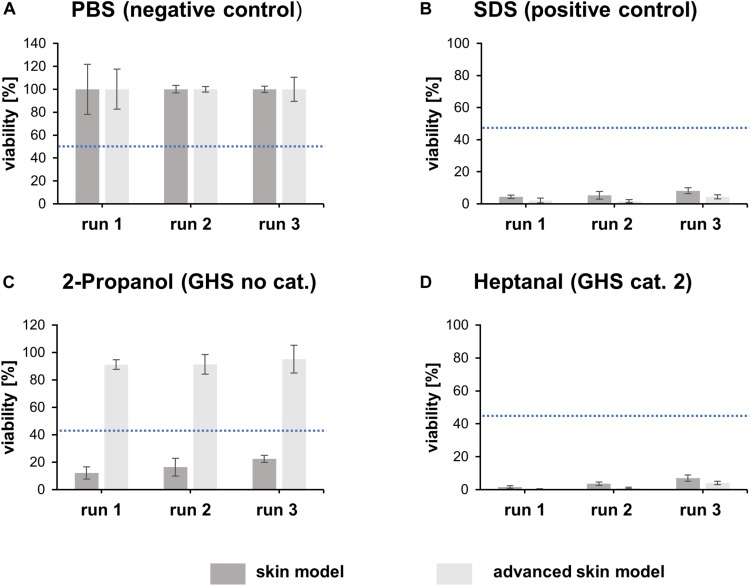
Irritation test with heptanal, SDS, 2-propanol and PBS. The bar graphs represent the percentual viability of skin models/advanced skin models after treatment with PBS **(A)**, SDS **(B)**, 2-propanol **(C)**, and heptanal **(D)**. Treatment with PBS was set as negative control and treatment with SDS as positive control. All values were normalized to the negative control (set to 100%). Mean values are displayed with SD. Dashed blue lines highlight the threshold of 50% viability compared to the negative control (*n* = 3).

The irritation potential of substances is commonly detected by cell viability, after the topical application of the substance and a subsequent post-incubation time. A negative (PBS) and positive control (5% SDS solution) were included in every test run. The viability was assessed using a WST assay. Tissue viability of the negative control was set to 100% (Figure 6A). The positive control demonstrates the sensitivity of the tissue model to a known irritant. The mean viability of the positive control was always clearly below the threshold of 50% as defined in the prediction model (Figure 6B).

To identify the predictive capacity of the models, the *in vitro* classification obtained was compared to the reference *in vivo* UN GHS classifications. Both skin models classified the irritating substance heptanal (GHS cat. 2) correctly (Figure 6D). However, the traditional skin model misclassified 2-propanol (GHS no cat.) as irritating whereas the advanced skin model classified the substance correctly as non-irritating (Figure 6C).

**FIGURE 7:**
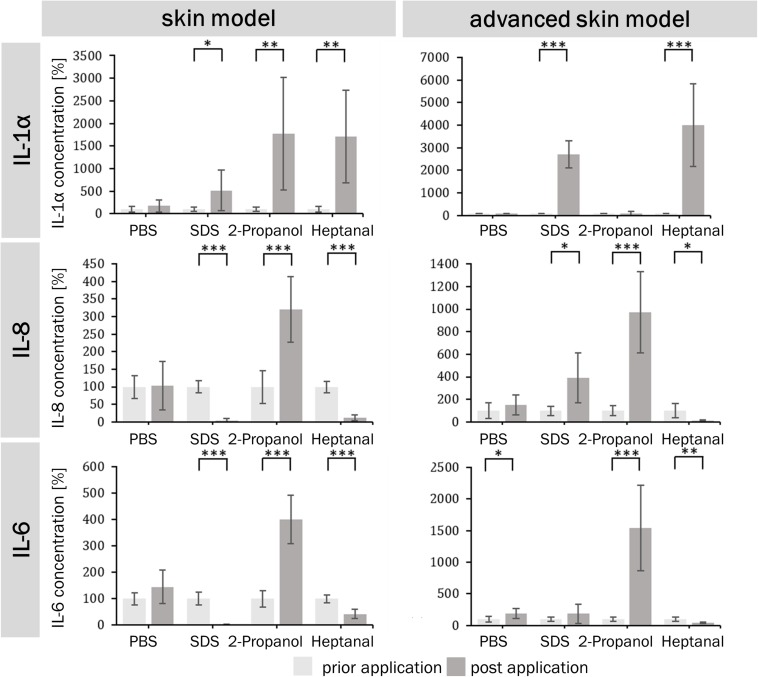
Percentual IL-1α, IL-6, and IL-8 levels prior and post application of irritating and non-irritating substances. Bar graphs depict percentual concentration values of the cytokines IL-1α, IL-6, and IL-8 for of the skin model **(A,C,E)** and the advanced skin model **(B,D,F)** normalized to prior application of the different substances (light gray). Cytokines were detected by ELISA in the supernatants of the respective skin models 48 h after the last media change prior and post application of substances (*n* = 3). PBS served as negative control and SDS as positive control. Level of significance ^∗^*p* < 0.05, ^∗∗^*p* < 0.01, ^∗∗∗^*p* < 0.001.

The potential of a skin irritation reaction could be correlated to the release of cytokines (IL-1α, IL-6, and IL-8). The extracellular cytokine release/secretion of IL-1α, IL-8, and IL-6 was measured prior to the substance exposure and after the post-exposure phase of 48 h (Figure 7). The negative control had limited influence on the cytokine release in both skin models. The positive control lead to an increase in IL-1α secretion in both models, a decrease in IL-8 and IL-6 secretion in the traditional skin model, and an increase in IL-8 for the advanced model. When the models were treated with the strong irritant substance heptanal, both showed a significant increase in IL-1α secretion and a significant decrease in IL-8 and IL-6 secretion. The treatment of 2-propanol lead to an increase of IL-1α only in the traditional skin model, while the advanced skin model showed no reaction to the treatment. However, both test systems showed an increase of IL-8 and IL-6 following incubation with 2-propanol.

## Discussion

For the generation of *in vitro* skin models, accurately reproducing the different layers and components of human skin is critical. The simultaneous culture of different cell types is a difficult process, which has yet to be optimized. The use of collagen I hydrogels for the production of full thickness skin equivalents reflects closely human tissue, as the extracellular component of the connective tissue mainly consist of collagen I and III. However, the collagen hydrogels undergo a fibroblast-mediated contraction. The use of chemical modifications to avoid the contraction alters the cell behavior and leads to a reduced viability or/and inhibits cell proliferation (Lotz et al., 2017). The new approach we have established in this study has no influence on cell behavior as no unnatural cross-linking or toxic substances were used to alter the hydrogel construction. Since the rearrangement of existing collagen fibrils and synthesis of a new matrix by fibroblasts is a physiological process, its prevention is undesirable. Another approach uses self-assembled dermal equivalents, where isolated human fibroblasts produce their specific fibroblast-derived matrix (Berning et al., 2015). However, this process lasts around 4 weeks, followed by an epidermal differentiation of at least 14 days. This leads to high costs and minimizes the use as a high-throughput system, necessary in industry and clinical contexts. The standard skin models experienced a strong contraction during 15 days of culture, visible in a remaining surface area of 17%. These weak mechanical properties, caused by the strong fibroblast-mediated shrinkage of collagen gels, have already been reported by other research groups (El-Ghalbzouri et al., 2002; Boehnke et al., 2007; Lotz et al., 2017; Schimek et al., 2018). This diminishes the suitability of skin models as a test system for topically applied substances, as they do not cover the whole surface and the substance under investigation can openly diffuse through the insert membrane (Ackermann et al., 2010; Groeber et al., 2011). The advanced skin model developed in this study showed no reduction of the epidermal surface area due to shrinkage, sealing the complete insert surface and modeling a skin barrier more accurately. However, the epidermal part is separated from the dermis because keratinocytes were seeded into the insert directly on the PET membrane which functions as an artificial basal membrane.

A main function of human skin is to serve as a non-permeable barrier between the body and the environment (Elias, 2007). The barrier function of skin models is often assessed by topical application of substances, a suitable route for drug delivery (Naik et al., 2000; Torin Huzil et al., 2011). The presence of an intact skin barrier is also associated with resistance to chemicals (Mathes et al., 2014). The function of the barriers in skin models is crucial and should be tested by using substances that penetrate the skin such as fluorescein sodium and FITC-dextran or impair barrier structure such as Triton-X (El Ghalbzouri et al., 2008). The human skin is permeable for small molecules with molecular weights of <500 g/mol (Abd et al., 2016).FITC-dextran (4000 g/mol) showed no permeation through the advanced skin model whereas fluorescein sodium (∼380 g/mol) is able to permeate to a low extend (17% after 6 h of incubation), demonstrating the suitability of this *in vitro* model compared to currently available skin models. These findings attest an intact barrier function of the advanced skin model. The resistance of the skin models and advanced skin models to detergent application was tested by topical Triton-X application. Both skin models experienced a significant reduction in viability measured by WST-1 assay compared to untreated skin models. However, the advanced skin model maintained 53% viability and the skin model only 25% after 1.5 h of detergent application. This indicates a higher resistance of the advanced skin model to Triton X-100 penetration compared to the skin model, which demonstrated superiority in the advanced skin model permeability.

Human skin has a complex multilayered structure. Especially the formation of a stratified epidermis with the different layers, namely basal, spinous, granular and cornified layer comprising keratinocytes with distinct morphology and protein expression pattern, is critical for skin models and their barrier function (Baroni et al., 2012; Kumar and Jagannathan, 2018). No effects on epidermal differentiation and the building of the stratum corneum were observed. More over our model shows a comparable histological architecture to human skin. Taken together we developed a new method to generate volume stable 3-layered skin models showing typical morphological characteristics with a functional barrier for further testing.

*In vitro* skin models represent a valuable tool for substance testing in the cosmetic industry. Since the ban of animal testing for cosmetic research, the need and the requirement for skin models have increased significantly (SCCS, 2006). The OECD defines in its guideline 439 requirements which skin models must met in order to be used in a standardized manner for the evaluation of skin irritation (OECD, 2019b). The performance standards state the need of necessary test methods, essential reference chemicals and must reliably and accurately define the results generated with the respective skin models (Liebsch et al., 2011). In this study, the OECD TG 439 was used as a basis to compare the traditional and advanced skin models in their ability to correctly classify an irritating and a non-irritating substance, as determined by viability after irritant exposure. The commonly used generation of skin models led to a misclassification of 2-propanol, whereas the advanced set-up classified both substances correctly. Misclassification of substances in *in vitro* irritation testing was previously reported in other studies where reconstructed epidermal models were used (Alepee et al., 2010; Jung et al., 2014; Groeber et al., 2016; Mewes et al., 2016). However, 2-propanol was never misclassified in other studies using epidermal models. However, the OECD acceptance criterion of ≥80% sensitivity, ≥70% specificity and ≥75% accuracy, doesn’t require a correct classification of all 20 reference substances in a validation study. None-the-less, it can be assumed that if 2-propanol is classified incorrectly, other substances will also be misclassified. The shrinkage of the epidermis leads to an impaired connection between the insert wall and the epidermis, which is highly accountable for the misclassification of 2-propanol. The next step should be the testing of all 20 reference chemicals defined in the OECD performance standards for *in vitro* skin irritation testing.

Pro-inflammatory cytokines such as IL-1α, IL-6, and IL-8 are involved in skin reaction to irritants. Therefore, extracellular cytokine release can be used to define the irritation potential of substances. IL-1α is an important inflammatory mediator in the skin located in keratinocytes, where it is constitutively expressed and accumulates in the cytoplasm or as a membrane bound form (Dinarello, 1998; Grone, 2002). It is only released from cells with disrupted membranes, e.g., after detergent application, which results in the release of the cytoplasm (Osborne and Perkins, 1994). This correlates with the IL-1α concentrations of both skin models, which were elevated after irritant exposure. The skin model also showed increased IL-1α concentrations after 2-propanol application, which is consistent with the false classification of 2-propanol as irritating. An increase of IL-6 and IL-8 after 2-propanol exposure occurred for both models. These results demonstrate that these cytokines could be used to show the outcome of mild irritating effects, which cannot be detected in viability studies.

In conclusion, an advanced three-layered skin model suitable as *in vitro* test system for irritating substances was successfully established. Our *in vitro* test system enables the topical application of substances, as well as the analysis of the influence on adipose tissue and the introduction of new endpoints like drug deposition would be possible. This improved model is groundbreaking, in how it overcomes current obstacles of alternative methods for animal testing.

## Data Availability Statement

The datasets generated for this study are available on request to the corresponding author.

## Ethics Statement

All research was carried out in accordance with the rules for investigation of human subjects as defined in the Declaration of Helsinki. Patients gave a written agreement according to the permission of the Landesärztekammer Baden-Württemberg (F-2012-078; for normal skin from elective surgeries).

## Author Contributions

FS and PK conceived and presented the idea, and supervised the findings of this work. FS and SN designed the study and analyzed the data. SN performed the experiments. All authors discussed the results and contributed to the final manuscript.

## Conflict of Interest

The authors declare that the research was conducted in the absence of any commercial or financial relationships that could be construed as a potential conflict of interest.
